# A case of toxic epidermal necrolysis with extensive intestinal involvement

**DOI:** 10.1186/2045-7022-4-S3-P14

**Published:** 2014-07-18

**Authors:** Keiko Nishimura, Riichiro Abe, Makiko Yamaguchi, Takamasa Ito, Shinichi Nakazato, Youhei Hamade, Nao Saito, Reine Moriuchi, Takehiko Katsurada, Mika Watanabe, Maroto Maria Iitani, Hiroshi Shimizu

**Affiliations:** 1 Hokkaido University Graduate School of Medicine, Department of Dermatology, Japan; 2 Hokkaido University Graduate School of Medicine, Department of Gastroenterology, Japan; 3 JR Sapporo Hospital, Department of Dermatology, Japan

## 

Toxic epidermal necrolysis (TEN) is life-threatening cutaneous adverse drug reaction characterized by extensive keratinocyte death. Gastrointestinal (GI) involvement in TEN is rare, however it contributes to poor prognosis. Here, we report a case of TEN with extensive intestinal involvement. A 56-year-old female was treated by zonisamide for fibromyalgia. Ten days later, generalized erythema appeared with blister, superficial punctate keratitis, and severe diarrhea. Skin biopsy specimen revealed that marked keratinocyte death. She was diagnosed with Stevens-Johnson syndrome (SJS). In spite of methylprednisolone pulse therapy for three days and plasma exchange three times, the bullous lesions spread to cover 60% of the body surface area. She was finally diagnosed with TEN, and treated with high-dose intravenous immunoglobulins for three days and another methylprednisolone pulse therapy. After these therapies, skin lesions were gradually improved. Despite skin manifestation improvement, the patient's diarrhea persisted (2000ml/day), resulting in hypoalbuminemia (0.8g/dL). Upper endoscopy revealed extensive erosions in esophagus and duodenum. Colonoscopy revealed multiple erosions and edematous structures in distal ileum and colon. Biopsy specimens of ileum and colon showed extensive mucosal detachment and granulation tissues. Examinations of Clostridium difficile toxin and cytomegalovirus antigen were negative. According to these findings, we considered her diarrhea to be consistent with GI lesions related in TEN. We treated her with mucoprotective agent, total parenteral nutrition management, albumin supplementation, and tapered systemic corticosteroid slowly. GI symptoms were gradually improved. Approximately 15 cases of SJS/TEN with GI manifestations have been reported in English literature. The mortality rate of SJS/TEN with GI manifestations is about 45%. There is no specific treatment, and treatment has largely been supportive. GI symptoms are often delayed, as in our case. GI mucosal damage may result in enteral nutrition difficulty, hypoalbuminemia, poor wound healing and bacterial translocation. The prolonged intestinal lesions may be attributable to difficulties with re-epithelization of intestinal mucosa by digestive fluid and formation of granulation tissue. Intestinal involvement of SJS/TEN is refractory and life-threatening complication. Regardless of the severity of skin manifestations, we should carefully monitor the GI symptoms.

